# Mendelian randomization study shows a causal effect of asthma on chronic obstructive pulmonary disease risk

**DOI:** 10.1371/journal.pone.0291102

**Published:** 2023-09-01

**Authors:** Yuanyuan Li, Weina Wang, Dengfeng Zhou, Qiaofa Lu, Lili Li, Bo Zhang

**Affiliations:** 1 Department of Respiratory and Critical Care Medicine, Wuhan Fourth Hospital, Wuhan, Hubei Province, China; 2 Department of Gastroenterology, Wuhan Fourth Hospital, Wuhan, Hubei Province, China; NIPSOM: National Institute of Preventive and Social Medicine, BANGLADESH

## Abstract

**Background:**

This study was performed to explore the causal association between asthma and chronic obstructive pulmonary disease(COPD).

**Methods:**

We obtained summary statistics for asthma from 408,442 Europeans in an open genome-wide association study (GWAS) from the UK Biobank to select strongly associated single nucleotide polymorphisms that could serve as instrumental variables for asthma (P < 5×10^−8^). Additional summary statistics for COPD were obtained from 193,638 individuals of European ancestry in the GWAS published by FinnGen. Univariable Mendelian randomization(UVMR) analysis was performed using inverse variance weighted (IVW) as the primary method of analysis. The reliability of the results was verified by multivariable MR(MVMR), reverse and replication MR analysis, and sensitivity analysis.

**Results:**

In the UVMR analysis, asthma increased the risk of COPD, with an odds ratio (OR) of 1.27 (95% confidence interval (CI) = 1.16–1.39, P = 5.44×10^−7^). Estimates were consistent in MVMR analyses by the adjustments of smoking initiation, age of smoking initiation, cigarettes per day, PM 2.5, and the combination of the above factors. In the reverse MR analysis, there was no evidence of a causal effect of COPD on asthma risk(OR = 1.02, 95% CI = 0.97–1.07, P = 0.3643). In the replication MR analysis, asthma still increased the risk of COPD. Sensitivity analyses validated the robustness of the above associations.

**Conclusions:**

We found that genetically predicted asthma was positively associated with the risk of COPD. Additionally, there was no evidence that COPD increases the risk of asthma. Further clarification of this link and underlying mechanisms is needed to identify feasible measures to promote COPD prevention.

## Introduction

Chronic obstructive pulmonary disease (COPD) is a preventable condition characterized by persistent airflow restriction and airway symptoms [1]. In 2019, approximately 3.23 million people died from complications of COPD or COPD itself. COPD is the 3rd leading cause of death and the 7th leading cause of ill health in the world in terms of disability-adjusted life years (DALYs) [2,3]. Due to an aging population and environmental factors, COPD will pose a greater threat to human health and a heavier burden on public healthcare systems in the coming years.

Smoking has traditionally been considered a major cause of COPD. However, previous studies by Pena and colleagues have shown that a quarter of COPD cases occur in never-smokers in the United States, with similar proportions reported in the United Kingdom (22.9%) and Spain (23.4%). Estimates suggest that 25–45% of COPD patients have never smoked, indicating that the burden of COPD on non-smokers is much higher than previously thought. A growing number of published studies suggest that, in addition to smoking, other risk factors such as air pollution, infectious diseases, occupational exposure, and chronic asthma are strongly associated with COPD [4]. For instance, a prospective observational study found that adults with active asthma have a 12.5 times higher risk of developing COPD compared to non-asthma subjects, after adjusting for smoking history and other potential confounders [5]. Nearly a quarter of COPD patients present with a history of asthma, conversely, suffering from asthma is prone to develop COPD in smokers [6,7]. A further study reveals that asthma is an independent risk factor associated with COPD, without a link to smoking status [8]. A grouping number of studies have demonstrated the correlation between asthma and COPD, with most studies suggesting that asthma increases the incidence of COPD [9]. However, these observational studies are easily influenced by confounding and reverse causation, and it remains unclear whether the presence of asthma causally raises the risk of COPD.

Mendelian randomization (MR) is a genetic epidemiology method that uses genetic variants to estimate the causal effect of a specific factor on a disease or outcome. It is based on the assumption that genetic variants associated with a disease are randomly distributed in the population and are therefore independent of other confounding factors. This allows researchers to examine the relationship between a factor and a disease without the influence of confounding variables, which can lead to biased estimates [10–12]. Recent MR studies have confirmed the causality between asthma and gastrointestinal disorders [13], rheumatoid arthritis [14], and periodontitis [15]. Although previous observational studies have demonstrated an association between asthma and COPD, inferring a causal relationship can be challenging. This study aimed to assess the causal relationship between asthma and COPD through MR using publicly available summary statistics, providing a basis for controlling asthma and thus reducing the incidence of COPD.

## Materials and methods

### Study design

**Fig 1:**
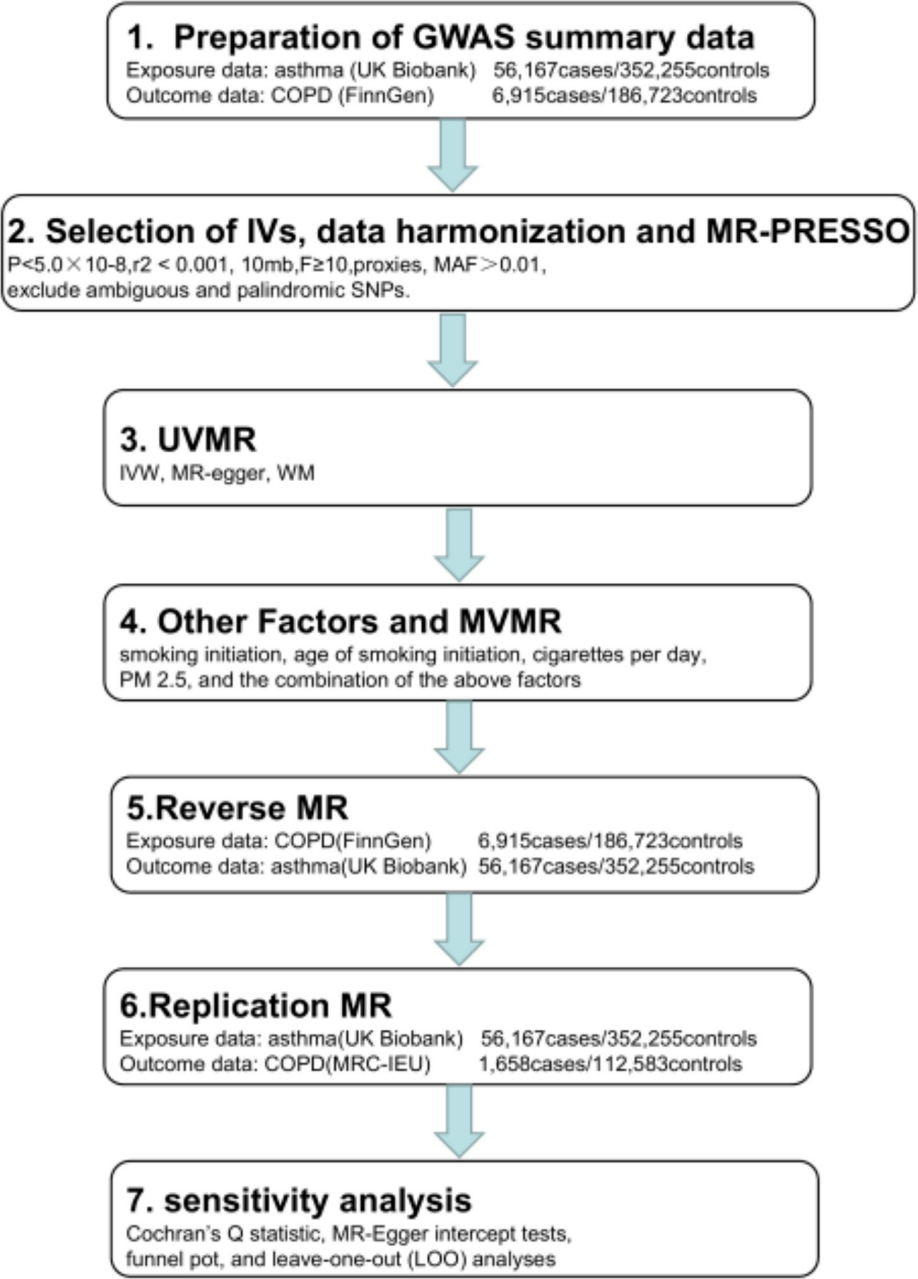
Analysis process of our research.

Using summary statistics from genome-wide association studies (GWAS), we conducted two-sample MR analyses to estimate the causal effects of asthma on COPD risk. Instrumental variables (IVs) were selected based on three assumptions: IVs must be strongly associated with the exposure of interest; IVs must be independent of unmeasured confounders; and IVs must affect the outcome only through the exposure of interest and not via confounders [16]. In this MR study, asthma and COPD were treated as exposure and outcome, respectively. We first performed univariable MR (UVMR) to explore the causal relationship between asthma and COPD, and then used multivariable MR (MVMR) to further assess the direct effect of asthma on COPD, adjusting for potential confounders (e.g. smoking initiation, age of smoking initiation, cigarettes per day, and PM 2.5) [1]. Additionally, we conducted reverse and replication MR analyses to validate the relationship. A flowchart of the whole procedure is shown in Fig 1. This study did not require separate ethical approval.

### Data sources

The summary statistics of asthma were from the latest large-scale GWAS meta-analysis of 408,442 Europeans (56,167 cases and 352,255 controls) from the UK Biobank, including results from association test of 35,551,291 single nucleotide polymorphisms (SNPs) with asthma in a cohort of White British ancestry [17]. Additionally, summary statistics for COPD phenotypes were derived from the GWAS publicly available in the FinnGen database, including a total of 16,380,382 SNPs from 6,915 individual cases and 186,723 controls of European ancestry.

### Selection of IVs

In this MR study, SNPs were identified as meaningful at the genome-wide significance level (P<5.0×10^−8^), and those without linkage disequilibrium (LD) with other SNPs (r^2^ < 0.001 within a 10Mb clustering window) were used as IVs. We calculated the F-statistic to quantify the strength of genetic variation and eliminated SNPs with an F-statistic less than 10, which indicated insufficient strength [18]. For the missing SNPs in the resulting GWAS dataset, proxies were identified at the cut-off of R^2^ > 0.8 (https://snipa.helmholtz-muenchen.de/snipa3

/index.php). If no appropriate proxy was available, SNPs were dropped. SNPs with minor allele frequency (MAF)≤0.01 were removed. Harmonizing processes were conducted to exclude ambiguous and palindromic SNPs. The SNPs that were clearly correlated with results (P<5.0×10^−8^) were excluded. Finally, MR-pleiotropy residual sum and outlier (MR-PRESSO) tests were conducted prior to each MR analysis. These tests are particularly suitable for identifying horizontal polymorphisms in less than 50% of instruments, thereby eliminating potential outliers [19].

### UVMR

We carried out an MR analysis to estimate the causal effect of asthma on COPD risk. An inverse variance weighted (IVW) estimation method was used in the main analyses, combining each SNP with the resulting Wald ratio to obtain a pooled causality estimate. This method allows for overdispersion [20]. Moreover, other MR analyses, like MR-Egger regression, and weighted median, were implemented to supplement IVW and provide more reliable estimates in a wider range of situations.MR-Egger regression can test for multinomial and considerable heterogeneity of imbalances, but for the same degree of under-exposure variation, it requires a larger sample size[21]. In cases where at least half of the weighted variance provided by the horizontal pleiotropy is valid, the weighted median method delivers consistent effect estimates [22]. The two-sided P<0.05 was considered statistically significant.

### Other factors and MVMR

To further eliminate the potential for multiple effects, we manually searched through all IVs in Phenoscanner (http://www.phenoscanner.medschl.cam.ac.uk) to determine if they were associated with previously reported confounders(P < 5 × 10^−8^), such as smoking, air pollution, infections, occupational exposures, and immune dysfunction, among others. We found IVs related to smoking traits, which are closely associated with COPD [1]. We excluded these IVs of confounders in an additional model and re-analyzed the MR estimates to gain a more direct cause-effect association. Furthermore, MVMR was also conducted to minimize the correlated pleiotropy introduced by these confounders [23]. In MVMR, genetic signatures for smoking traits were obtained from a large GWAS, comprising up to 1.2 million European-descent participants, which included three smoking phenotypes: smoking initiation (n = 607,291), cigarettes per day(n = 337,334), and age of smoking initiation(n = 341,427) [24]. The summary genetic data on PM2.5 were obtained from the UK Biobank GWAS, which included 423,796 European participants. The study was based on the ESCAPE project (European Study of Cohorts for Air Pollution Effects), which used the LUR model to estimate PM2.5 pollution concentrations at the home addresses of study participants [25].

### Reverse MR analysis

We also performed a reverse MR analysis to explore reverse causality. Significant reverse MR analysis indicated reverse causality from exposure (COPD) to outcome (asthma). The procedure for reverse MR analysis is the same as the MR analysis above.

### Replication MR analysis

To validate the robustness, we replicated the IVW analysis using another independent COPD GWAS data from the MRC Integrative Epidemiology Unit (MRC-IEU). Genetic variants associated with COPD from the MRC-IEU consortium, which contained 1,658 COPD patients, 112,583 controls, as well as 9,851,867 SNPs.

### Sensitivity analysis

Sensitivity analysis in MR studies has been crucial to detect potential genetic polymorphisms and the heterogeneity of MR estimates. Therefore, we further performed Cochran’s Q statistic, MR-Egger intercept tests, funnel pot, and leave-one-out (LOO) analyses to detect the presence of pleiotropy and assess the robustness of the results. Cochran’s Q test (P <0.05 indicates heterogeneity) and I^2^ statistic (I^2^ value > 50% indicates heterogeneity) were adopted to assess the heterogeneity among SNPs in IVW estimates. A value of P< 0.05 indicated significant heterogeneity in which case a random-effect model was applied to subsequent analyses [22,26–28]. All of the analyses were run by using the R package TwoSampleMR (version 0.5.6) in R (version 4.1.1).

This study was reported in line with the Strengthening the Reporting of Observational Studies in Epidemiology using MR (STROBE-MR) guidance, with the checklist available in the Supporting information (S1 Table) [29].

## Results

### UVMR and sensitivity analysis

**Fig 2:**
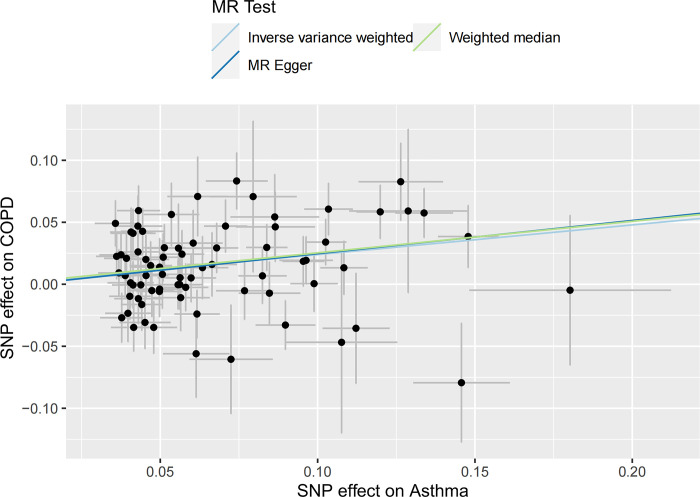
Scatter plot for the causal effect of asthma on the risk of COPD.

**Fig 3:**
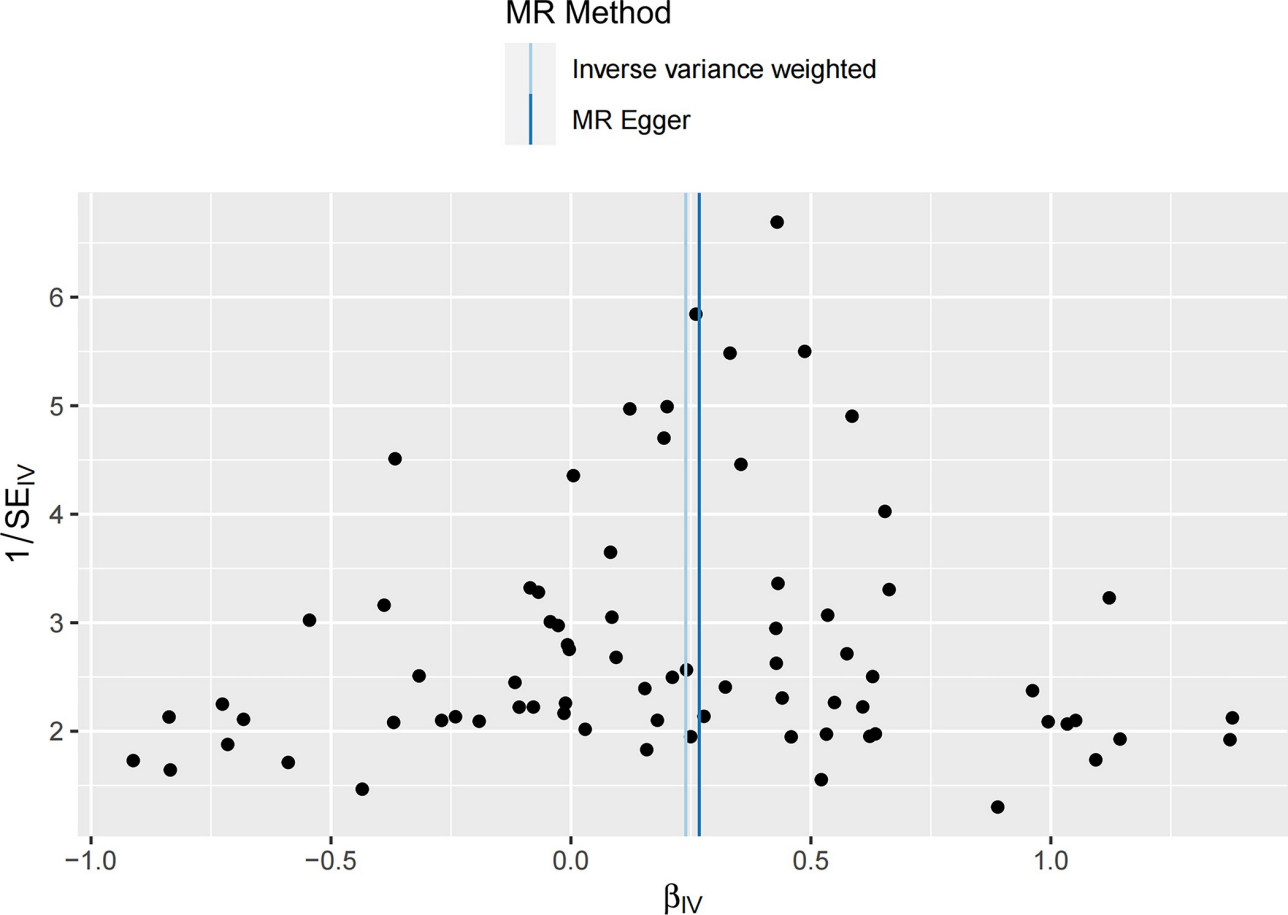
Funnel plot for the causal effect of asthma on the risk of COPD.

**Fig 4:**
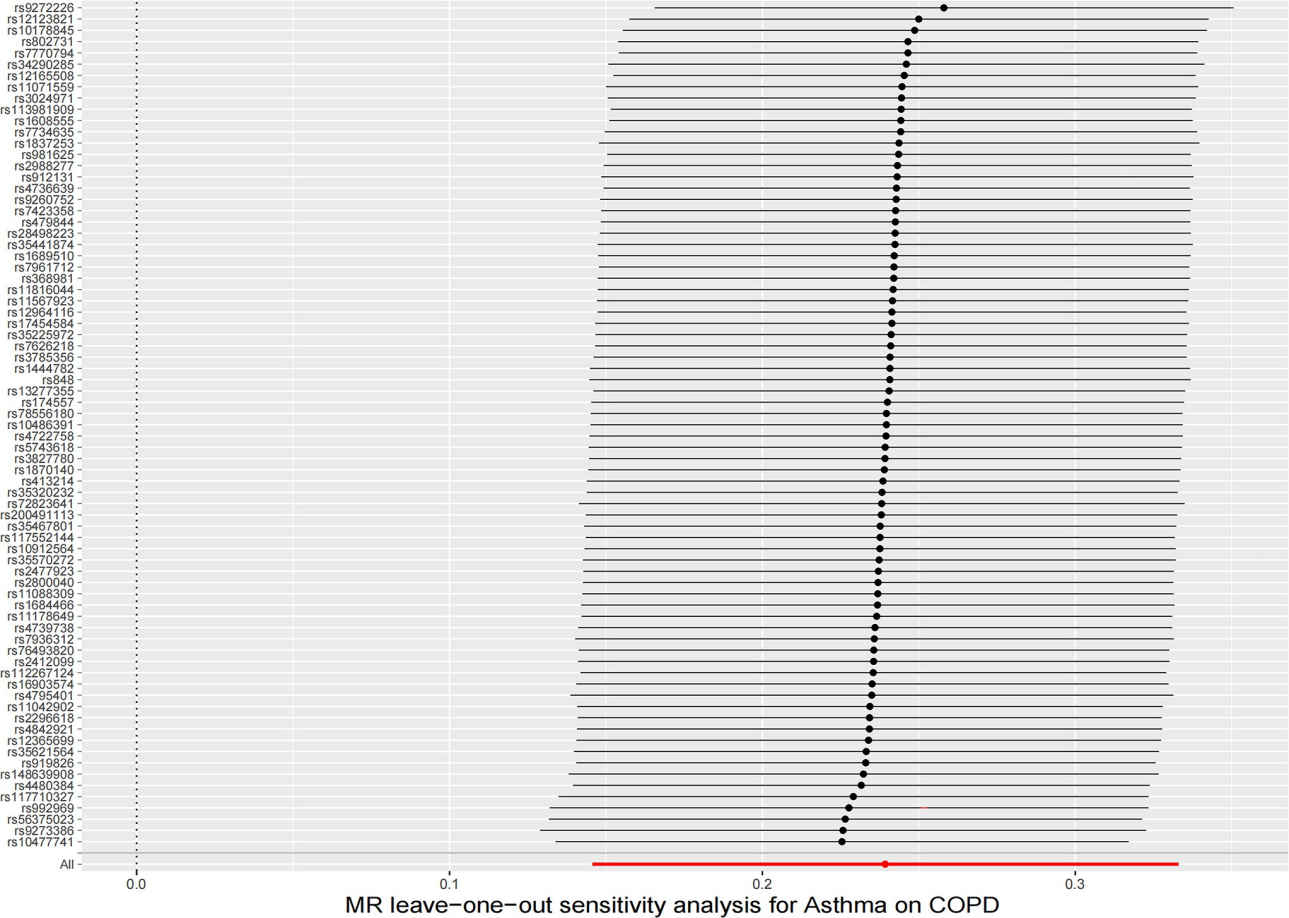
Leave-one-out analysis for the causal effect of asthma on the risk of COPD.

In the UVMR analysis, a total of 75 IVs were associated with the risk of COPD after a series of quality control steps. All F-values for inclusion of SNPs >10. As shown in Table 1, the primary results of IVW showed that asthma was positively associated with the risk of COPD (odds ratio (OR) = 1.27, 95% confidence interval (CI) = 1.16–1.39, P = 5.44×10^−7^). Meanwhile, similar risk estimates were obtained using the MR‐Egger regression (OR = 1.31, 95% CI = 1.03‐1.66, P = 0.0315) and weighted median approaches (OR = 1.29, 95% CI = 1.15‐1.45, P = 2.43×10^−5^) (Fig 2). The heterogeneity test detected some possible heterogeneity between individual SNP effect estimates(P = 0.0019) and therefore we choose the results of the IVW random effects. The funnel plot was symmetrical (Fig 3). LOO analysis also showed that the effect estimates were unaltered by any one variant (Fig 4). In addition, the MR-Egger regression intercept did not have significant evidence of horizontal pleiotropy.

**Table 1:** Causal relationships between asthma and COPD risk performed by MR.

Methods	Exposure	Outcome	Method	SNPs	Beta	P	OR	95%CI	Heterogeneity			Pleiotropy
									Q	Q_df	Q_p	egger_intercept_p
**UVMR**	Asthma	COPD	IVW	75	0.2393	5.44×10^−7^	1.27	(1.16,1.39)	114.22	74	0.0019	0.8030
	Asthma	COPD	MR Egger	75	0.2673	0.0315	1.31	(1.03,1.66)				
	Asthma	COPD	WM	75	0.2537	2.43×10^−5^	1.29	(1.15,1.45)				
**Reverse MR**	COPD	Asthma	IVW	3	0.0220	0.3643	1.02	(0.97,1.07)	1.51	2	0.4700	0.8023
	COPD	Asthma	MR Egger	3	0.0577	0.7035	1.06	(0.85,1.33)				
	COPD	Asthma	WM	3	0.0246	0.3711	1.02	(0.97,1.08)				
**Replication analysis**	Asthma	COPD	IVW	48	0.0061	3.33×10^−6^	1.01	(1.00,1.01)	52.95	47	0.2555	0.1747
	Asthma	COPD	MR Egger	48	0.0104	0.0034	1.01	(1.00,1.02)				
	Asthma	COPD	WM	48	0.0059	0.0011	1.01	(1.00,1.01)				

IVW, inverse variance weighted; WM, weighted median; UVMR, Univariable MR; MR, Mendelian randomization; SNPs, single nucleotide polymorphisms.

### Other factors and MVMR

Several factors may affect the association between asthma and the risk of COPD. To check the potential influence of confounding factors, we searched the Phenoscanner V2 website for the identified IVs with each type of association. Only one SNP (rs12165508) of asthma was associated with current smoking-related phenotypes. After removing the one SNP, the causality remained significant (IVW OR = 1.28, 95% CI = 1.16–1.40, P = 2.30×10^−7^). In the MVMR analysis, IVW estimates were consistent by the adjustments of smoking initiation (OR = 1.23, 95% CI = 1.11–1.36, P = 6.03×10^−5^), cigarettes per day (OR = 1.24, 95% CI = 1.10–1.39, P = 3.43×10^−4^), age of smoking initiation (OR = 1.26, 95% CI = 1.14–1.39, P = 9.09×10^−6^), PM 2.5 (OR = 1.26, 95% CI = 1.15–1.39, P = 1.34×10^−6^) and the combination of the above factors (OR = 1.22, 95% CI = 1.10–1.35, P = 1.19×10^−4^) (Table 2).

**Table 2:** Multivariable inverse variance weighted estimates for adjusted associations with COPD.

**Exposure**	Adjustment	SNPs	P	OR	95%CI
**Asthma**	smoking initiation	64	6.03×10^−5^	1.23	(1.11,1.36)
**Asthma**	Cigarettes per Day	65	3.43×10^−4^	1.24	(1.10,1.39)
**Asthma**	Age Of Smoking Initiation	67	9.09×10^−6^	1.26	(1.14,1.39)
**Asthma**	PM 2.5	70	1.34×10^−6^	1.26	(1.15,1.39)
**Asthma**	Age Of Smoking Initiation/Cigarettes per Day/smoking initiation/PM 2.5	62	1.19×10^−4^	1.22	(1.10,1.35)

COPD, chronic obstructive pulmonary disease; SNPs, single nucleotide polymorphisms; OR, odds ratio; CI, confidence interval.

### Reverse and replication MR analysis

In the reverse MR analysis, there was no evidence of a causal effect of COPD on asthma risk(OR = 1.02, 95% CI = 0.97–1.07, P = 0.3643) (Table 1). To further verify our results, replication MR analysis was conducted using COPD GWAS data from the MRC-IEU consortium. As expected, similar trends were observed using FinnGen COPD GWAS data ((IVW OR = 1.01, 95% CI = 1.00–1.01, P = 3.33×10^−6^) (Table 1). Sensitivity analysis suggested that the results were stable.

## Discussion

We performed a two-sample Mendelian randomization analysis to comprehensively assess whether asthma has a causal effect on the incidence of COPD, and the results suggested a causal relationship between genetically predicted asthma and increased risk of COPD.

MVMR, reverse and replication MR analyses, and sensitivity analyses further confirmed the statistical robustness of these findings. To our knowledge, this is the first MR study to systematically assess the causal role of asthma in COPD issues.

Asthma and COPD are caused by different mechanisms triggered by different pathogens, but some patients present with features of both asthma and COPD, which is known as asthma-COPD overlap syndrome (ACOS). ACOS may have several different underlying mechanisms. Orie et al. proposed airway hyperreactivity as a genetic condition and a common risk factor for the development of asthma and COPD [30]. Genetic analysis associated with the development of asthma and COPD has established several disease susceptibility genes such as ADAM33, and orosomucoid-like 3, which are frequent for both asthma and COPD [31,32]. Viral infections in early childhood are a risk factor for the development of asthma and COPD [33]. Various factors associated with early life events associated with asthma and COPD from the fetal stage to early childhood have been associated with impaired airway and lung growth and the subsequent onset of asthma and COPD [34]. The pathogenesis of COPD due to asthma is currently unknown but may involve airway remodeling, chronic airway inflammation, and bronchoconstriction independent of inflammation. The histological characteristics of airway remodeling in patients with asthma-induced COPD include thickening of the bronchial epithelial basement membrane, increase in airway smooth muscle mass, increase in mucus-producing cells, subepithelial fibrosis, and vascularization [35]. In addition, a randomized controlled trial showed that allergen-induced bronchoconstriction and methotrexate-induced bronchoconstriction contribute to persistent airflow obstruction in asthma patients by up-regulating transforming growth factor beta and epithelial-mesenchymal transition [36].

Our finding on the association between asthma and COPD risk is consistent with previous observational studies. A 50-year cohort study in Aberdeen demonstrated childhood asthma significantly increases the risk of COPD (OR = 6.37, 95%CI = 3.73–10.94) [37]. Similar findings have been observed in other cohort studies [5,7,38]. In addition, several cross-sectional studies also provide some supporting evidence [8,39–43]. The Wellington Respiratory Survey, which involved 749 individuals aged between 25 and 75 years to investigate the risk factors of COPD, revealed that a prior diagnosis of asthma was the most frequently associated risk factor [39]. The CanCOLD study, a multicenter cross-sectional investigation, revealed self-reported asthma was an independent predictor of COPD in both never-smokers and ever-smokers [8]. Further, a systematic review and modeling analysis including 162 articles documenting population-based studies conducted in 260 study centers across 65 countries showed that asthma significantly increases the risk of developing COPD, with an OR of 2.6 (95%CI = 1.6–4.1) in the global countries, and a higher risk observed in low and middle-income countries (OR = 3.6, 95%CI = 0.5–24.1) [44]. However, these types of study designs may fail to distinguish between causality and non-directional associations caused by confounding factors and are prone to errors of reverse causality.

One strength of this study is that the results obtained in MR studies can strengthen the evidence for causal inference. As an IV, SNP overcomes environmental influence and disease development, and can effectively avoid reverse causality and confounding bias. The large-scale GWAS data used in the study for MR analysis can improve the accuracy of the results. However, we cannot ignore several limitations of this MR study. First, due to the lack of data in the original study, such as gender, asthma phenotype, and asthma severity, further subgroup analyses have been difficult to perform. Further research is needed to explore the relationship between asthma and COPD in different subpopulations, especially those with specific asthma phenotypes or severity. Second, we used MVMR to check for the possibility of horizontal pleiotropy introduced by the observed confounders. However, multivariate analysis cannot overcome bias due to pleiotropic effects from pathways outside of smoking or PM 2.5. Third, although the Mendelian randomization method provides us with a way of estimating causal relationships between genes and phenotypes, we cannot be completely certain of the causal relationship between asthma and COPD due to the limitations of the effect size and the number of SNPs. Finally, our MR analysis included only the European population, so we need more evidence from other populations to draw a confirmed conclusion.

In conclusion, asthma could increase the risk of COPD, while there is no evidence that COPD increases the risk of asthma. More research is needed in the future to investigate the underlying mechanisms, such as functional studies, genomics studies, and multi-omics studies. Meantime, clinicians should pay attention to the risk of COPD in asthmatic patients and formulate early diagnosis and treatment strategies for these patients.

## Supporting information

S1 Table STROBE-MR checklist of recommended items to address in reports of Mendelian randomization studies. (DOCX)
